# CX3CL1 promotes MMP-3 production via the CX3CR1, c-Raf, MEK, ERK, and NF-κB signaling pathway in osteoarthritis synovial fibroblasts

**DOI:** 10.1186/s13075-017-1487-6

**Published:** 2017-12-21

**Authors:** Sheng-Mou Hou, Chun-Han Hou, Ju-Fang Liu

**Affiliations:** 1 Department of Orthopedic Surgery, Shin Kong Wu Ho-Su Memorial Hospital, No. 95, Wen Chang Road, Taipei, 111 Taiwan; 2 Department of Orthopedic Surgery, National Taiwan University Hospital, No. 1, Jen-Ai Road, Taipei, 100 Taiwan; 3 Central Laboratory, Shin-Kong Wu Ho-Su Memorial Hospital, No. 95, Wenchang Road, Shilin, Taipei, 111 Taiwan

**Keywords:** CX3CL1, CX3CR1, Osteoarthritis, Matrix metalloproteinase 3

## Abstract

**Background:**

Osteoarthritis (OA) is a degenerative joint disease that affects the cartilage, synovium, and subchondral bone and is the leading cause of disability in older populations. Specific diagnostic biomarkers are lacking; hence, treatment options for OA are limited. Synovial inflammation is very common in OA joints and has been associated with both OA’s symptoms and pathogenesis. Confirming the role of the synovium in OA pathogenesis is a promising strategy for mitigating the symptoms and progression of OA. CX3CL1 is the only member of the CX3C class of chemokines that combines the properties of chemoattractants and adhesion molecules. CX3CL1 levels in the synovium and serum were both discovered to be positively associated with OA pathogenesis. CX3CL1 and its receptor CX3CR1 belong to a family of G protein-coupled receptors. Matrix metalloproteinases (MMPs), which are responsible for matrix degradation, play a crucial role in OA progression. The relationship between CX3CL1 and MMPs in the pathophysiology of OA is still unclear.

**Methods:**

CX3CL1-induced MMP-3 production was assessed with quantitative real-time PCR and ELISA. The mechanisms of action of CX3CL1 in different signaling pathways were studied using western blot analysis, quantitative real-time PCR and ELISA. Neutralization antibodies of integrin were achieved to block the CX3CR1 signaling pathway. Luciferase assays were used to study NF-κB promoter activity.

**Results:**

We investigated the signaling pathway involved in CX3CL1-induced MMP-3 production in osteoarthritis synovial fibroblasts (OASFs). CX3CL1 was found to induce MMP-3 production in a concentration-dependent and time-dependent manner. Using pharmacological inhibitors and CX3CR1 small interfering RNA to block CX3CR1 revealed that the CX3CR1 receptor was involved in the CX3CL1-mediated upregulation of MMP-3. CX3CL1-mediated MMP-3 production was attenuated by c-Raf inhibitors (GW5074) and MEK/ERK inhibitors (PD98059 and U0126). The OASFs were stimulated using CX3CL1-activated p65 phosphorylation.

**Conclusions:**

Our results demonstrate that CX3CL1 activates c-Raf, MEK, ERK, and NF-κB on the MMP-3 promoter through CX3CR1, thus contributing to cartilage destruction during OA.

## Background

Osteoarthritis (OA), a common progressive degenerative disease, is the most frequent cause of physical disability, which affects more than 12.4 million individuals aged 65 years and older. The prevalence of OA in the United States is estimated to increase by approximately 9 million from 1995 to 2020 [1]. The etiology of OA is currently unclear. Its main pathological characteristics are cartilage loss, change in the subchondral bone, and thickening of the synovium [2]. The goals of OA therapy are joint pain reduction and joint function improvement. The available strategies for preventing or treating OA are limited. The normal synovial membrane comprises an intimal lining one or two cell layers thick. A typical feature of OA is synovial lining hyperplasia, which increases the number of synovial fibroblasts (SFs) [3]. These OASFs are a source of proinflammatory cytokines and proteolytic enzymes, including matrix-degrading enzymes (matrix metalloproteinases (MMPs) and aggrecanases), which contribute to articular matrix degradation [4–8]. Therefore, elucidating the molecular mechanisms of OA can facilitate the development of novel anti-OA strategies.

Human chemokines are divided into four families (C, CC, CXC, and CX3C) depending on the conserved cysteine motif. Chemokines are chemoattractant proteins that regulate leukocyte trafficking, inflammation, and immune responses. Numerous studies have established a correlation between chemokine expression and inflammatory diseases including arthritis, atherosclerosis, asthma, and metabolic syndrome. CX3CL1 is expressed in many cell types, including neurons, intestinal epithelium, and activated vascular endothelium, and is structurally distinct from other chemokines. Several studies have indicated that CX3CL1 plays a central role in inflammatory diseases. In 2002, Cockwell et al. [9] discovered that CX3CL1 expression was increased in acute human renal inflammation. In 2003, Ollivier et al. [10] reported that CX3CL1 triggered not only monocyte adhesion but also chemotactic function and was involved in the pathogenesis of atherosclerosis. CX3CR1 is a seven-transmembrane domain G protein-coupled receptor and the specific receptor for CX3CL1. CX3CR1 mediates several intracellular signaling pathways, such as the p38MAPK signaling pathway [11] and the Akt pathway [12]. Several pieces of evidence have suggested that CX3CL1–CX3CR1 interactions contribute to the development of inflammatory diseases such as rheumatoid arthritis (RA) [13, 14]. CX3CL1 is overexpressed in the serum, synovium, synovial fluid, and cartilage of patients with RA [14, 15].

CX3CL1 may also promote MMP-2 production in SFs [16]. This confirms the role of CX3CL1 in the pathogenesis of OA; however, the molecular connections between CX3CL1 and OA remain largely elusive. Therefore, we explored the signaling pathways involved in CX3CL1-induced MMP-3 production in human OASFs in addition to the role of CX3CL1 in the pathogenesis of OA to determine whether CX3CL1 is an appropriate target for drug intervention in OA in the future.

## Methods

### Cell culture

Written informed consent was obtained from all patients recruited into this study, and the study was approved by the Institutional Review Board of Shin Kong Wu Ho-Su Memorial Hospital. Synovial tissue was obtained from patients with OA, and SFs were isolated. Human SFs were isolated by collagenase treatment of synovial tissue samples obtained from 10 patients with OA during knee-replacement surgeries and eight samples of nonarthritic synovial tissues obtained at arthroscopy after trauma/joint derangement. Fresh synovial tissues were finely minced and digested in Dulbecco’s modified Eagle’s medium (DMEM) containing 2 mg/ml type II collagenase (Sigma-Aldrich, St. Louis, MO, USA) for 4 h at 37 °C and under 5% CO_2_. The isolated cells were placed in DMEM containing 10% fetal bovine serum (FBS), 100 units/ml penicillin, 100 μg/ml streptomycin, and 2 mM l-glutamine at 37 °C with 5% CO_2_. Passages 4–6 of the obtained OASFs were used in this study. Results of four independent experiments are presented [4, 17].

### Materials

DMEM, Lipofectamine3000, and Trizol were purchased from Invitrogen (Carlsbad, CA, USA). Cell culture dishes, FBS, six-well plates, and 12-well plates were purchased from Corning (Corning, NY, USA). Polyvinyldifluoride (PVDF) membranes and an Immobilon Western Chemiluminescent HRP Substrate detection system were purchased from Millipore (Billerica, MA, USA). Polyclonal antibodies specific for MMP3, CX3CL1, CX3CR1 and IKKα/β were purchased from Santa Cruz Biotechnology (Santa Cruz, CA, USA). Monoclonal antibodies specific for c-Raf, MEK, ERK, IκBα, p65, and β-Actin were purchased from Santa Cruz Biotechnology (Santa Cruz, CA, USA). Polyclonal rabbit antibodies specific for c-Raf phosphorylated at Ser338 and IKKα/β phosphorylated at ser176/180 were purchased from Cell Signaling and Neuroscience (Danvers, MA, USA). Monoclonal rabbit antibodies specific for MEK1/2 phosphorylated at Ser217/221, ERK1/2 phosphorylated at Thr202/204, IκBα phosphorylated at ser32/36, and p65 phosphorylated at ser536 were purchased from Cell Signaling and Neuroscience (Danvers, MA, USA).3-(3,5-Dibromo-4-hydroxybenzyliden)-5-iodo-1,3-dihydroindol-2-one (GW5074), 1,4-diamino-2,3-dicyano-1,4-bis(*o*-aminophenylmercapto)butadiene monoethanolate (U0126), 2-(2-amino-3-methoxyphenyl)-4H-1-benzopyran-4-one (PD98059), pyrrolidine dithiocarbamate (PDTC), and l-1-tosylamido-2-phenylenylethyl chloromethyl ketone (TPCK) were purchased from Sigma-Aldrich. Recombinant human CX3CL1 was purchased from PeproTech (Rocky Hill, NJ, USA). The small interfering RNA (siRNA) of the control and CX3CR1 siRNA were purchased from Santa Cruz Biotechnology. Nuclear factor kappa B (NF-κB) luciferase plasmid was purchased from Stratagene (La Jolla, CA, USA). A pSV-β-galactosidase vector and a luciferase assay kit were purchased from Promega (Madison, MA, USA). All other chemicals were purchased from Sigma-Aldrich.

### RNA extraction and quantitative real-time polymerase chain reaction

Total RNA was extracted from cells using Trizol reagent (Invitrogen) following the manufacturer’s protocol. In brief, cells were added to 0.5 ml Trizol, homogenized, and incubated at room temperature for 3 min. After extraction with chloroform (0.1 ml) and precipitation with isopropanol (0.4 ml), RNA was washed with 75% ethanol, and finally the RNA pellet was dissolved in 10 μl of RNase-free water. The RNA yield and purity were determined by measuring absorbance at 260 and 280 nm using a Nanodrop spectrophotometer (Thermo Fisher Scientific, Inc., Waltham, MA, USA). RNA was then used to synthesize complementary DNA (cDNA) using reverse transcriptase (Invitrogen) according to the manufacturer’s instructions.

Real-time quantitative polymerase chain reaction (qPCR) was performed using SYBR Green (KAPA Biosystems, Woburn, MA, USA) according to the manufacturer’s protocol, and reactions were performed using a StepOnePlus machine (Applied Biosystems, Foster City, CA, USA). Human MMP-1, MMP-2, MMP-3, MMP-7, MMP-9, MMP-12, MMP-3, and glyceraldehyde 3-phosphate dehydrogenase (GAPDH) purchased from Sigma-Aldrich were used as primers to amplify the target genes. The expression levels of the target genes were determined by normalizing them to the GAPDH levels. We calculated the results using the following equation:

$$ \mathrm{Ratio}={2}^{\hbox{-} \Delta \Delta \mathrm{C}\mathrm{t}},\kern1em \mathrm{where}\  \Delta \Delta \mathrm{C}\mathrm{t}={\left(\mathrm{C}{\mathrm{t}}_{\mathrm{t}\mathrm{arget}}\hbox{-} \mathrm{C}{\mathrm{t}}_{\mathrm{GADPH}}\right)}_{\mathrm{Sample}}\hbox{-} {\left(\mathrm{C}{\mathrm{t}}_{\mathrm{t}\mathrm{arget}}\hbox{-} \mathrm{C}{\mathrm{t}}_{\mathrm{GADPH}}\right)}_{\mathrm{Control}}. $$

Each sample was assayed in triplicate, and the data represent three independent experiments.

### Western blot analysis

Cellular lysates were prepared using the methods outlined in previous studies. Proteins were resolved using sodium dodecyl sulfate polyacrylamide gel electrophoresis and transferred to Immobilon PVDF membranes. The blots were blocked with 5% BSA for 1 h at room temperature and then probed using antihuman antibodies against MMP-3, CX3CL1, CX3CR1, c-Raf, MEK, ERK, IKKα/β, IκB, p65, and β-Actin (1:1000) for 1 h at room temperature. After three washes, the blots were incubated with secondary antibodies (1:10,000) for 1 h at room temperature. The blots were then visualized using a charge-coupled device camera-based detection system (UVP Inc., Upland, CA, USA). Quantitative data were obtained using ImageJ software (National Institute of Health, USA).

### Determination of MMP-3 secretions using enzyme-linked immunosorbent assay

MMP-3 in the cell culture supernatants was then determined using a Quantikine enzyme-linked immunosorbent assay (ELISA) kit (R&D Systems, Minneapolis, MN, USA) according to the manufacturer's protocol. In brief, OASFs were seeded in 100-mm culture dishes at a density of 5 × 10^6^ cells per dish and then treated under different conditions. Following 24 h of incubation, the culture supernatant was collected and centrifuged at 10,000 rpm for 10 min and stored at −80 °C in fresh tubes.

### Transfection and luciferase receptor activity

Transfection was performed using Lipofectamine 3000 transfection reagent (LF3000; Invitrogen) according to the manufacturer’s instructions. Cells were transfected with control siRNA, CX3CR1 siRNA, vector, dominant negative MEK mutants, dominant negative ERK mutants, dominant negative IKKα mutants, dominant negative IKKβ mutants, and luciferase plasmid using Lipofectamine 3000 in optiMEM medium. After 24 h of transfection, cells were incubated with the indicated agents. After 24 h of incubation, the luciferase activity in the transfected cells was measured using a Luciferase Reporter Assay System (Promega) according to the manufacturer’s instructions. Transactivation was determined by monitoring the firefly luciferase levels in the pGL2 vector. The luciferase assay was performed by adding lysis buffer (100 μl) and harvesting the cells through centrifugation (13,000 rpm for 5 min). The supernatant was transferred to fresh tubes, and 20 μl of cell lysate was added to 80 μl of fresh luciferase assay buffer in an assay tube. The luciferase activity was measured using a microplate luminometer. Luciferase activity was normalized to transfection efficiency based on the cotransfected β-galactosidase expression vector.

### Chromatin immunoprecipitation assay

Crosslinked chromatin was prepared from OASF cells, and a chromatin immunoprecipitation (ChIP) assay was performed using a Pierce Magnetic ChIP kit (Thermo Fisher Scientific, Inc.) according to the manufacturer’s protocol. After immunoprecipitation with anti-p65 antibody or control IgG, protein A/G magnetic beads were added. DNA was purified and analyzed using PCR. The following MMP-3 primers were used: 5′-AATTCACATCACTGCCACCA-3′ (forward) and 5′-CTCTGTGGCAATAAGATCCC-3′ (reverse).

### Statistics

Values are reported as mean ± standard error of the mean (SEM). A statistical comparison between two samples was performed using the Student *t* test. Statistical comparisons of more than two groups were performed using one-way analysis of variance with the Bonferroni post-hoc test. In all comparisons, *p* < 0.05 was considered significant.

## Results

### CX3CL1-induced MMP-3 production in human OASFs

**Fig. 1:**
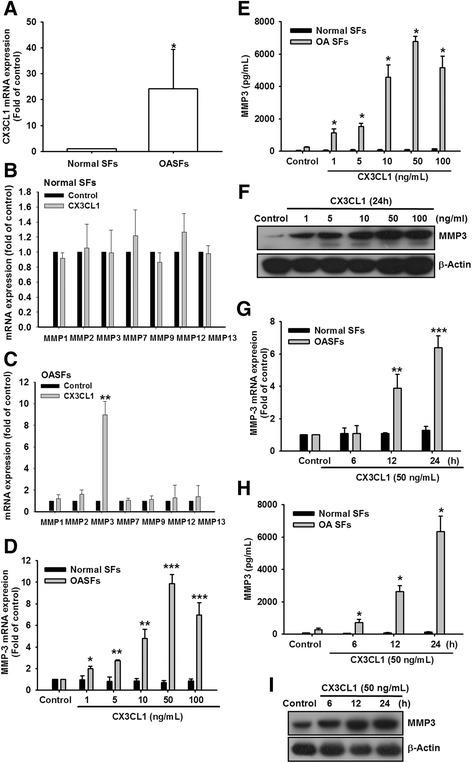
Concentration-dependent and time-dependent increases in MMP-3 production by CX3CL1. **a** Human SFs were obtained from healthy patients (*n* = 8) or patients with OA (*n* = 10). CX3CL1 expression examined using qPCR. **b**, **c** OASFs and normal SFs were incubated with CX3CL1 (50 ng/ml) for 24 h. mRNA expression of MMPs examined using qPCR (*n* = 4). **d**, **g** OASFs and normal SFs were incubated with various concentrations of CX3CL1 for 24 h or with CX3CL1 (50 ng/ml) for 6, 12, or 24 h. mRNA expression of MMP-3 examined using qPCR (*n* = 4). **e**, **h** OASFs and normal SFs were incubated with various concentrations of CX3CL1 for 24 h or with CX3CL1 (50 ng/ml) for 6, 12, or 24 h; supernatants and cell lysates were then collected. MMP-3 level in culture media measured using a Quantikine ELISA kit (*n* = 4). **f**, **i** MMP-3 protein levels in cell lysates determined using western blot analysis. Both protein levels and enzymatic activity increased in a dose-dependent and time-dependent manner. Results expressed as mean ± SEM. * represents P < 0.05, ** represents P < 0.01, ***represents P < 0.001, as compared to respective control by using one-way ANOVA followed by Bonferroni's post-hoc test.. MMP matrix metalloproteinase, OASF osteoarthritis synovial fibroblast

CX3CL1 is known to participate in the pathogenesis of OA and RA pathogenesis [14, 18]. Therefore, we first compared the CX3CL1 levels in normal human SFs (normal SFs) and OASFs. The mRNA expression of CX3CL1 was higher in the OASFs than in the normal SFs (Fig. 1a). Because CX3CL1 stimulates MMP expression in chronic liver diseases [19], we hypothesized that any of these MMPs could be involved in CX3CL1-directed OA pathogenesis. We used qPCR to detect mRNA expression levels of MMPs in normal SFs and OASFs. The expression of MMP-3 was significantly higher than that of other MMPs in OASFs compared with the basal level expressed in normal SFs (Fig. 1b, c). To understand the relationship between CX3CL1 and MMP-3 in normal SFs and OASFs, we examined the level of MMP-3 after CX3CL1 treatment. The level of MMP-3 was significantly elevated in OASFs compared with normal SFs. CX3CL1 induced MMP-3 production in a concentration-dependent manner (Fig. 1d–f), and induction occurred in a time-dependent manner in OASFs (Fig. 1 g–i). These results indicated that CX3CL1 increased MMP-3 production in human OASFs.

### CX3CL1–CX3CR1 interaction induced MMP-3 expression in OASFs

**Fig. 2:**
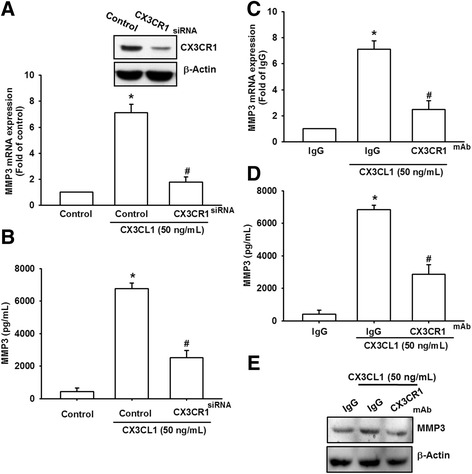
CX3CR1 is involved in CX3CL1-mediated MMP-3 production in OASFs. **a**, **b** OSAFs were transfected for 24 h with CX3CR1 siRNA, followed by stimulation with CX3CL1 for 24 h. MMP-3 expression examined using qPCR and ELISA. **c**-**e** OASFs were pretreated for 30 min with CX3CR1 mAb followed by stimulation with CX3CL1 for 24 h. MMP-3 expression was examined using qPCR, ELISA and western blot. Results expressed as mean ± SEM (*n* = 3). *p < 0.05 compared with control; #*p* < 0.05 compared with CX3CL1-treated group. mAb monoclonal antibody, MMP matrix metalloproteinase, siRNA small interfering RNA

The CX3CL1–CX3CR1 axis plays a crucial role in the development of inflammatory diseases [10, 20]. Therefore, we hypothesized that CX3CR1 is involved in CX3CL1-induced MMP-3 production. We knocked down CX3CR1 expression by transfecting the OASFs with CX3CR1 siRNA and determined that CX3CR1 siRNA inhibited CX3CL1-induced MMP-3 production at the mRNA and protein expression levels (Fig. 2a, b). Furthermore, a CX3CL1 monoclonal antibody (mAb), but not the control IgG, effectively suppressed CX3CL1-induced MMP-3 mRNA and protein expression (Fig. 2c–e). These results suggest that CX3CR1 activation may be responsible for CX3CL1-induced MMP-3 expression.

### CX3CL1-induced MMP-3 production through the c-Raf/MEK/ERK pathway

**Fig. 3:**
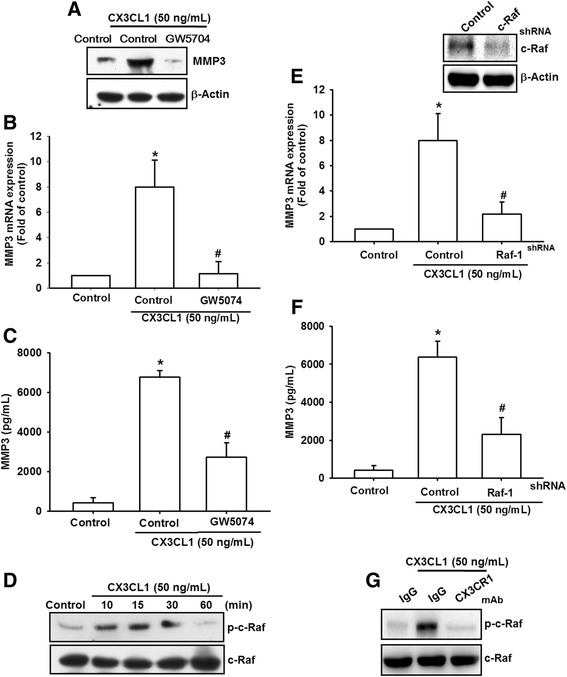
c-Raf is involved in CX3CL1-mediated MMP-3 production in SFs. **a** OASFs were incubated with CX3CL1 for the indicated time intervals. c-Raf phosphorylation examined using western blot analysis. **b** OASFs were pretreated for 30 min with CX3CR1 mAb followed by stimulation with CX3CL1 for 15 min. c-Raf protein levels in the cell lysates determined using western blot analysis. **c**–**e** OASFs were pretreated for 30 min with c-Raf inhibitor (GW5074, 10 μM), followed by stimulation with CX3CL1 for 24 h. MMP-3 expression examined using qPCR, western blot analysis, and ELISA. **f**, **g** OASFs were transfected for 24 h with c-Raf shRNA followed by stimulation with CX3CL1 for 24 h. MMP-3 expression examined using qPCR and ELISA. Results expressed as mean ± SEM (*n* = 3). *p < 0.05 compared with control; #*p* < 0.05 compared with CX3CL1-treated group. MMP matrix metalloproteinase, shRNA small hairpin RNA

To examine the mechanism by which CX3CL1 induces MMP-3 production, we directly measured the c-Raf phosphorylation in response to CX3CL1. The results revealed that the stimulation of cells using CX3CL1 induced c-Raf phosphorylation in a time-dependent manner (Fig. 3a). Pretreatment of cells with the CX3CR1 antibody attenuated c-Raf phosphorylation, suggesting that CX3CR1 serves as the upstream regulator of c-Raf-mediated signaling (Fig. 3b). Furthermore, to examine whether CX3CL1 stimulates the production of MMP-3 through c-Raf signaling, we used pharmacological inhibitor (GW5704) and c-Raf shRNA. Pretreatment of cells with GW5074 was found to antagonize CX3CL1-induced MMP-3 production at the mRNA and protein levels (Fig. 3c–e). As shown in Fig. 3f, g, CX3CL1-induced MMP-3 production at the mRNA and protein levels was strongly reduced in shRNA against c-Raf.

**Fig. 4:**
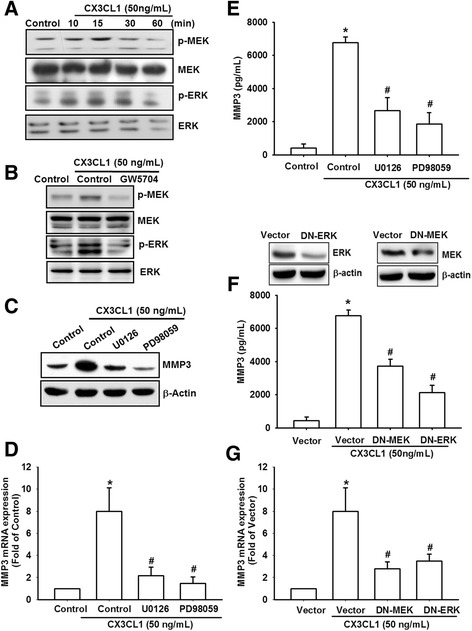
MEK/ERK is involved in CX3CL1-mediated MMP-3 production in OASFs. **a** OASFs were incubated with CX3CL1 for the indicated time intervals. MEK/ERK phosphorylation examined using western blot analysis. **b** OASFs were pretreated for 30 min with c-Raf inhibitor (GW5074) followed by stimulation with CX3CL1 for 15 min. MEK/ERK protein levels in the cell lysates determined using western blot analysis. **c**–**e** OASFs were pretreated for 30 min with MEK/ERK inhibitors (U0126 and PD98059) followed by stimulation with CX3CL1 for 24 h. MMP-3 expression examined using qPCR, western blot analysis, and ELISA. **f**, **g** OASFs were transfected for 24 h with MEK and ERK mutants followed by stimulation with CX3CL1 for 24 h. MMP-3 expression examined using qPCR and ELISA. Results expressed as mean ± SEM (*n* = 3). *p < 0.05 compared with control; #*p* < 0.05 compared with CX3CL1-treated group. MMP matrix metalloproteinase

Subsequently, we investigated whether CX3CL1 can activate MEK/ERK, which is a critical downstream target of c-Raf. Stimulation of cells with CX3CL1 was discovered to induce the time-dependent phosphorylation of MEK and ERK (Fig. 4a). However, this CX3CL1-induced phosphorylation of MEK/ERK was markedly decreased by inhibiting upstream signaling events using the c-Raf inhibitor (GW5704) (Fig. 4b). To further evaluate whether the MEK/ERK pathway can induce MMP-3 expression, we pretreated cells with PD98059 (10 μM) and U0126 (10 μM). CX3CL1 induced the mRNA and significantly reduced the protein levels of MMP-3 when cells were pretreated with PD98059 and U0126 (Fig. 4c–e). To further confirm this stimulation-specific mediation by MEK and ERK, we assessed the role of MEK and ERK using dominant negative mutations. Transfection of cells with dominant negative MEK and dominant negative ERK reduced MEK and ERK expression, respectively (Fig. 4f, upper panel). Transfection of cells with dominant negative MEK and dominant negative ERK effectively inhibited CX3CL1-induced MMP-3 mRNA and protein expression (Fig. 4e–g). These results indicate that CX3CL1 induces MMP-3 production through CX3CR1 activation, which consequently activates the c-Raf/MEK/ERK signaling pathways in OASFs.

### Involvement of NF-κB in CX3CL1-induced MMP-3 production

**Fig. 5:**
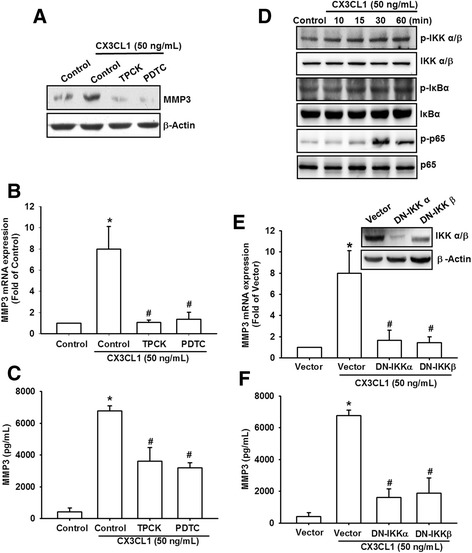
NF-κB is involved in the potentiation of MMP-3 production by CX3CL1. **a**–**c** OASFs were pretreated for 30 min with PDTC (10 μM) and TPCK (10 μM) followed by stimulation with CX3CL1 for 24 h. MMP-3 expression examined using qPCR, western blot analysis, and ELISA. **d** OASFs were incubated with CX3CL1 for the indicated time intervals. p-IKKα/β, p-IκBα, and p-p65 expression determined using western blot analysis. **e**, **f** OASFs were transfected for 24 h with IKKα and IKKβ mutants followed by stimulation with CX3CL1 for 24 h. MMP-3 expression examined using qPCR and ELISA. Results expressed as mean ± SEM (*n* = 3). *p < 0.05 compared with control; #*p* < 0.05 compared with CX3CL1-treated group. MMP matrix metalloproteinase, PDTC pyrrolidine dithiocarbamate, TPCK l-1-tosylamido-2-phenylenylethyl chloromethyl ketone

The activation of NF-κB can induce MMP-3 production in the cells of patients with RA or OA [21]. NF-κB is a transcriptional activator that plays a vital role in OA pathogenesis [22]. To examine whether NF-κB is involved in the signal transduction pathway leading to CX3CL1-induced MMP-3 production, we used NF-κB inhibitors (PDTC) and IκB protease inhibitors (TPCK). Pretreatment of cells with PDTC and TPCK was discovered to inhibit CX3CL1-induced MMP-3 mRNA and protein expression (Fig. 5a–c). We further examined the upstream molecules involved in CX3CL1-induced NF-κB activation. The stimulation of OASFs using CX3CL1 increased IKKα/β, IkBα, and p65 phosphorylation in a time-dependent manner (Fig. 5d). In addition, transfection of cells with IKKα and IKKβ mutants reduced CX3CL1-induced MMP-3 production and MMP-3 mRNA expression (Fig. 5e, f).

**Fig. 6:**
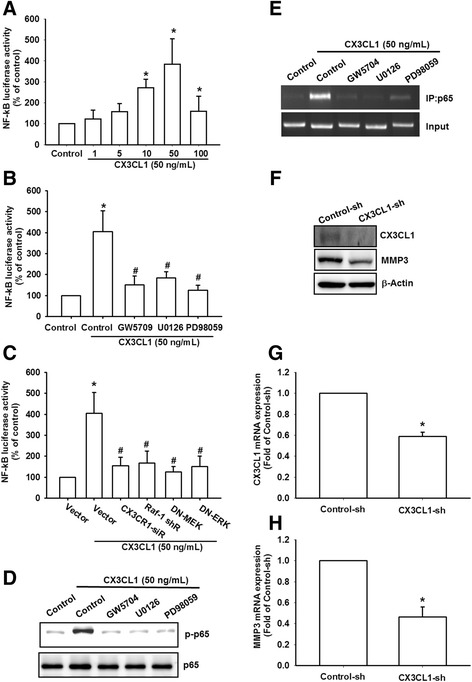
CX3CL1 induced NF-κB activation through the CX3CR1/c-Raf/MEK/ERK pathway. **a**–**c** OASFs were incubated with various concentrations of CX3CL1 or pretreated with c-Raf inhibitors (GW5074) or MEK/ERK inhibitors (U0126 and PD98059) for 30 min or transfected with c-Raf shRNA, MEK, ERK, IKKα, and IKKβ mutants before exposure to CX3CL1. NF-κB luciferase activity measured, and results normalized to the β-galactosidase activity. **d** OASFs were pretreated with c-Raf inhibitors (GW5074) or MEK/ERK inhibitors (U0126 and PD98059) for 30 min followed by stimulation with CX3CL1 for 60 min. p-p65 expression examined using western blot analysis. **e** Cells were pretreated with 0.1% dimethyl sulfoxide as control, c-Raf inhibitors (GW5074), or MEK/ERK inhibitors (U0126 and PD98059) for 30 min, followed by CX3CL1 treatment for 120 min. ChIP performed using an antibody against p65. One percent of immunoprecipitated chromatin was assayed to verify equal loading (input). **f**–**h** Protein and mRNA levels of CX3CL1 and MMP-3 in control-shRNA and CX3CL1-shRNA OASFs examined using western blotting and qPCR. Results expressed as mean ± SEM (*n* = 4). *p < 0.05 compared with control; #*p* < 0.05 compared with CX3CL1-treated group. MMP matrix metalloproteinase, NF-κB nuclear factor kappa B, sh short hairpin

To confirm that NF-κB is involved in CX3CL1-induced MMP-3 expression, we performed transient transfection using NF-κB promoter–luciferase constructs. When OASFs were incubated with CX3CL1, the NF-κB promoter activity increased in a dose-dependent manner (Fig. 6a). The increase in NF-κB activity induced by CX3CL1 was antagonized by c-Raf inhibitor (GW5704), MEK inhibitors (PD98059 and U0126), and c-Raf shRNA, MEK, ERK, IKKα, and IKKβ mutants (Fig. 6b, c). Furthermore, GW5704, PD98059, and U0126 reduced CX3CL1-mediated p65 phosphorylation (Fig. 6d). In addition, these inhibitors (Gw5074, PD98059, and U0126) reduced the CX3CL1-induced binding of p65 to an NF-κB element (Fig. 6e). To further investigate CX3CL1-mediated MMP-3 expression in OASFs, we established CX3CL1-shRNA expression cells. Western blot analyses were employed to compare the CX3CL1 expression levels in stable transfectants. CX3CL1 expression was drastically inhibited in OASF/CX3CL1-shRNA cells (Fig. 6f–h). In addition, CX3CL1 knockdown downregulated the expression of MMP-3 in OASFs (Fig. 6f–h). These data suggest that the CX3CR1, c-Raf, MEK, ERK, and NF-κB pathways must be activated if CX3CL1-induced MMP-3 production is to occur in human OASFs.

## Discussion

The present study provided compelling data to support the novel role of CX3CL1 in the severity of OA through its induction of MMP-3 production via the NF-κB pathway. Accumulating evidence suggests that CX3CL1 plays a vital role in the pathogenesis and progression of OA [14, 23]. In the present study, the CX3CL1 levels in OASFs were significantly higher than those in normal SFs (Fig. 1). Previous studies have demonstrated that patients with knee OA had significantly higher levels of serum, synovial fluid, and synovial CX3CL1 than is found in normal synovial fluid [24–26], which is consistent with the present study’s results. In addition, the role of CX3CL1 has been reported in inflammatory diseases [27]. CX3CL1 induces tumor necrosis factor alpha (TNF-α), interferon gamma, and interleukin 1 beta (IL-1β) production in chronic obstructive pulmonary disease, pulmonary hypertension, atherosclerosis, RA, HIV infection, and cancer [28–30]. In joint cartilage cells, the increase in CX3CL1 mRNA expression correlated with IL-1β [31]. Accumulating evidence suggests that CX3CL1 plays a more critical role in stimulating the inflammatory process in OA. The findings indicate that CX3CL1 may be considered a chemokine suitable for developing new therapeutic approaches for OA.

The MMP family comprises a group of zinc-ion-dependent endopeptidases that play an important role in normal and OA synovial tissue [32]. Unregulated MMP production results in excessive extracellular matrix degradation and leads to OA. MMP-3 (also known as stromelysin-1) is capable of degrading aggrecan and collagen types ***I,*** **II, III, IX, X and XI** in joints [33]. Accumulated evidence indicates that MMP-3 is not expressed in normal adult cartilage, but is highly expressed in the cartilage of patients with OA [34]. In addition, some studies have reported that CX3CL1 induces MMP production, including that of MMP-2 and MMP-9 [16, 35]. We identified MMP-3 as the target protein of the CX3CL1 signaling pathway, which regulates cartilage breakdown. CX3CL1 was discovered to induce MMP-3 mRNA and protein expression in a dose-dependent and time-dependent manner in OASFs. These results suggest that CX3CL1 acts as an inducer of MMPs and enhances cartilage breakdown.

A previous study indicated that the activation of CX3CR1 signaling may be a causal factor of OA [31]. The high expression of CX3CR1 in the synovial membranes of patients with OA may be directly involved in the pathophysiology of OA [31]. The results of our study indicated that CX3CL1 protein levels were significantly higher in OASFs than in normal SFs. We also discovered that CX3CR1 was required for CX3CL1-induced MMP-3 production. The incubation of cells with the CX3CR1 mAb inhibited CX3CL1-induced MMP-3 expression. In addition, CX3CR1 siRNA inhibited the increase in CX3CL1-induced MMP-3 production. These findings suggest that CX3CR1 is involved in CX3CL1-induced MMP-3 production in human OASFs.

In 2006, Lee et al. [36] demonstrated that the activation of CX3CL1 signaling in the c-Raf/MEK/ERK and PI3K/Akt/eNOS/NO signal pathways plays a vital role in molecular biological functions. However, the mechanisms for inducing MMP expression in different cell types may be regulated differently. The c-Raf/MEK/ERK signaling pathways that induce MMP expression in OASFs have not been reported. In this study, we demonstrated that the ability of CX3CL1 to induce MMP-3 production is mediated by the interaction between CX3CL1 and CX3CR1 and the subsequent activation of the c-Raf/MEK/ERK pathway. Our results demonstrated that treatment of OASFs with c-Raf inhibitor or transfection of cells with c-Raf shRNA reduced the CX3CL1-induced MMP-3 expression. However, we also found that CX3CL1 treatment increased the level of c-Raf phosphorylation. Moreover, the CX3CR1 antibody inhibited CX3CL1-mediated c-Raf phosphorylation. These results suggest that CX3CL1 induced MMP-3 production through CX3CR1 and the c-Raf signaling pathway in SFs. The activation of the MAPK pathway by G-coupled protein receptors generally involves c-Raf [37, 38]. In our experiments, GW0574 completely inhibited CX3CL1-induced MEK and ERK activation and MMP-3 production, suggesting that these effects of CX3CL1 require c-Raf activation.

Accumulating evidence suggests that MMP production is regulated by activation of the ubiquitous transcription factor NF-κB. In addition, the critical role of NF-κB in the pathophysiology of OA has been reported [39, 40]. Under normal conditions, the p65 subunit of NF-κB is retained in the cytoplasm with the inhibitory protein IκB; however, when NF-κB is activated by stimuli such as IL-1β or TNF-α, the phosphorylated p65 subunit of NF-κB translocates to the nucleus to regulate the expression of inflammatory mediators and MMPs [41, 42]. In the present study, we used NF-κB inhibitors to explore these pathways. We demonstrated that NF-κB activation contributed to CX3CL1-induced MMP-3 expression in human SFs. The pretreatment of cells with NF-κB inhibitors TPCK and PDTC reduced the CX3CL1-induced MMP-3 expression. Therefore, the NF-κB binding site is important in CX3CL1-induced MMP-3 production. The NF-κB sequence binds to members of the p65 and p50 families of transcription factors, and the results of this study revealed that CX3CL1 induced p65 phosphorylation and nuclear accumulation. Furthermore, the use of transient transfection with NF-κB-luciferase as an indicator of NF-κB activity revealed that CX3CL1 increased NF-κB activation. In addition, the c-Raf inhibitor (GW5074), MAPK inhibitors (U0126 and PD98059) or c-Raf shRNA or MEK, and the ERK mutant reduced CX3CL1-increased NF-κB promoter activity. These results indicate that CX3CL1 increases NF-κB activation through the CX3CR1/c-Raf/MAPK signaling pathway in human OASFs. The discovery of this CX3CL1 signaling pathway elucidates the mechanism underlying OA pathogenesis, which may lead to the development of effective therapies in the future.

## Conclusion

We explored the signaling pathways involved in CX3CL1-induced MMP-3 production in human SFs. We determined that CX3CL1 increases MMP-3 production by binding to CX3CR1 and activating c-Rad, MEK, and ERK signaling, which enhances NF-κB transcription activity and results in the transactivation of MMP-3 production. Furthermore, the discovery of CX3CL1/CX3CR1-mediated signaling pathways increases the understanding of the mechanism of OA pathogenesis and could facilitate the development of effective therapies for OA in the future.

## Abbreviations

cDNA: Complementary DNA
ChIP: Chromatin immunoprecipitation
DMEM: Dulbecco’s modified Eagle’s medium
ELISA: Enzyme-linked immunosorbent assay
FBS: Fetal bovine serum
GAPDH: Glyceraldehyde 3-phosphate dehydrogenase
mAb: Monoclonal antibody
MMP: Matrix metalloproteinase
NF-κB: Nuclear factor kappa B
OA: Osteoarthritis
OASF: Osteoarthritis synovial fibroblast
PVDF: Polyvinyldifluoride
qPCR: Real-time quantitative polymerase chain reaction
SEM: Standard error of the mean

## References

[1] von Bernstorff M, Feierabend M, Jordan M, Glatzel C, Ipach I, Hofmann UK (2017). Radiographic hip or knee osteoarthritis and the ability to drive. Orthopedics.

[2] Dziri C, Aloulou I, Loubiri I, Rekik M, Zohra Ben Salah F, Abdallah A (2016). Assessment of disability in osteoarthritis of the knee. Ann Phys Rehabil Med.

[3] Scanzello CR, Goldring SR (2012). The role of synovitis in osteoarthritis pathogenesis. Bone.

[4] Chen YT, Hou CH, Hou SM, Liu JF (2014). The effects of amphiregulin induced MMP-13 production in human osteoarthritis synovial fibroblast. Mediators Inflamm.

[5] Zeng GQ, Chen AB, Li W, Song JH, Gao CY (2015). High MMP-1, MMP-2, and MMP-9 protein levels in osteoarthritis. Genet Mol Res.

[6] Jiang Q, Qiu YT, Chen MJ, Zhang ZY, Yang C (2013). Synovial TGF-beta1 and MMP-3 levels and their correlation with the progression of temporomandibular joint osteoarthritis combined with disc displacement: a preliminary study. Biomed Rep.

[7] Felson DT (2006). Clinical practice. Osteoarthritis of the knee. N Engl J Med.

[8] Achari Y, Reno CR, Frank CB, Hart DA (2012). Carrageenan-induced transient inflammation in a rabbit knee model: molecular changes consistent with an early osteoarthritis phenotype. Inflamm Res.

[9] Cockwell P, Chakravorty SJ, Girdlestone J, Savage CO (2002). Fractalkine expression in human renal inflammation. J Pathol.

[10] Ollivier V, Faure S, Tarantino N, Chollet-Martin S, Deterre P, Combadiere C, de Prost D (2003). Fractalkine/CX3CL1 production by human aortic smooth muscle cells impairs monocyte procoagulant and inflammatory responses. Cytokine.

[11] Wu XM, Liu Y, Qian ZM, Luo QQ, Ke Y (2016). CX3CL1/CX3CR1 axis plays a key role in ischemia-induced oligodendrocyte injury via p38MAPK signaling pathway. Mol Neurobiol.

[12] Li D, Chen H, Luo XH, Sun Y, Xia W, Xiong YC (2016). CX3CR1-mediated Akt1 activation contributes to the paclitaxel-induced painful peripheral neuropathy in rats. Neurochem Res.

[13] Clark AK, Staniland AA, Malcangio M (2011). Fractalkine/CX3CR1 signalling in chronic pain and inflammation. Curr Pharm Biotechnol.

[14] Nanki T, Imai T, Kawai S (2017). Fractalkine/CX3CL1 in rheumatoid arthritis. Mod Rheumatol.

[15] Odai T, Matsunawa M, Takahashi R, Wakabayashi K, Isozaki T, Yajima N, Miwa Y, Kasama T (2009). Correlation of CX3CL1 and CX3CR1 levels with response to infliximab therapy in patients with rheumatoid arthritis. J Rheumatol.

[16] Blaschke S, Koziolek M, Schwarz A, Benohr P, Middel P, Schwarz G, Hummel KM, Muller GA (2003). Proinflammatory role of fractalkine (CX3CL1) in rheumatoid arthritis. J Rheumatol.

[17] Hou CH, Tang CH, Hsu CJ, Hou SM, Liu JF (2013). CCN4 induces IL-6 production through alphavbeta5 receptor, PI3K, Akt, and NF-kappaB singling pathway in human synovial fibroblasts. Arthritis Res Ther.

[18] Zhao L, Wang Q, Zhang C, Huang C. Genome-wide DNA methylation analysis of articular chondrocytes identifies TRAF1, CTGF, and CX3CL1 genes as hypomethylated in osteoarthritis. Clin Rheumatol. 2017; 36(10)2335-42. 10.1007/s10067-017-3667-9 28470428

[19] Bourd-Boittin K, Basset L, Bonnier D, L'Helgoualc'h A, Samson M, Theret N (2009). CX3CL1/fractalkine shedding by human hepatic stellate cells: contribution to chronic inflammation in the liver. J Cell Mol Med.

[20] Ferretti E, Pistoia V, Corcione A (2014). Role of fractalkine/CX3CL1 and its receptor in the pathogenesis of inflammatory and malignant diseases with emphasis on B cell malignancies. Mediators Inflamm.

[21] Tzeng HE, Chen JC, Tsai CH, Kuo CC, Hsu HC, Hwang WL, Fong YC, Tang CH (2011). CCN3 increases cell motility and MMP-13 expression in human chondrosarcoma through integrin-dependent pathway. J Cell Physiol.

[22] Imagawa K, de Andres MC, Hashimoto K, Pitt D, Itoi E, Goldring MB, Roach HI, Oreffo RO (2011). The epigenetic effect of glucosamine and a nuclear factor-kappa B (NF-kB) inhibitor on primary human chondrocytes—implications for osteoarthritis. Biochem Biophys Res Commun.

[23] Huo LW, Ye YL, Wang GW, Ye YG (2015). Fractalkine (CX3CL1): a biomarker reflecting symptomatic severity in patients with knee osteoarthritis. J Investig Med.

[24] Yano R, Yamamura M, Sunahori K, Takasugi K, Yamana J, Kawashima M, Makino H (2007). Recruitment of CD16+ monocytes into synovial tissues is mediated by fractalkine and CX3CR1 in rheumatoid arthritis patients. Acta Med Okayama.

[25] Klosowska K, Volin MV, Huynh N, Chong KK, Halloran MM, Woods JM (2009). Fractalkine functions as a chemoattractant for osteoarthritis synovial fibroblasts and stimulates phosphorylation of mitogen-activated protein kinases and Akt. Clin Exp Immunol.

[26] Zou Y, Li Y, Lu L, Lin Y, Liang W, Su Z, Wang X, Yang H, Wang J, Yu C (2013). Correlation of fractalkine concentrations in serum and synovial fluid with the radiographic severity of knee osteoarthritis. Ann Clin Biochem.

[27] Shimoda S, Harada K, Niiro H, Taketomi A, Maehara Y, Tsuneyama K, Kikuchi K, Nakanuma Y, Mackay IR, Gershwin ME (2010). CX3CL1 (fractalkine): a signpost for biliary inflammation in primary biliary cirrhosis. Hepatology.

[28] Jones BA, Beamer M, Ahmed S (2010). Fractalkine/CX3CL1: a potential new target for inflammatory diseases. Mol Interv.

[29] Zhang J, Patel JM (2010). Role of the CX3CL1-CX3CR1 axis in chronic inflammatory lung diseases. Int J Clin Exp Med.

[30] Xiong Z, Leme AS, Ray P, Shapiro SD, Lee JS (2011). CX3CR1+ lung mononuclear phagocytes spatially confined to the interstitium produce TNF-alpha and IL-6 and promote cigarette smoke-induced emphysema. J Immunol.

[31] Wojdasiewicz P, Poniatowski LA, Kotela A, Deszczynski J, Kotela I, Szukiewicz D (2014). The chemokine CX3CL1 (fractalkine) and its receptor CX3CR1: occurrence and potential role in osteoarthritis. Arch Immunol Ther Exp (Warsz).

[32] van den Bosch MH, Blom AB, van de Loo FA, Koenders MI, Lafeber FP, van den Berg WB, van der Kraan PM, van Lent PL. Brief Report: Induction of Matrix Metalloproteinase Expression by Synovial Wnt Signaling and Association With Disease Progression in Early Symptomatic Osteoarthritis. Arthritis Rheumatol. 2017; 69(10)1978-83. 10.1002/art.40206 28678406

[33] Ma JD, Zhou JJ, Zheng DH, Chen LF, Mo YQ, Wei XN, Yang LJ, Dai L (2014). Serum matrix metalloproteinase-3 as a noninvasive biomarker of histological synovitis for diagnosis of rheumatoid arthritis. Mediators Inflamm.

[34] Tong Z, Liu Y, Chen B, Yan L, Hao D. Association between MMP3 and TIMP3 polymorphisms and risk of osteoarthritis. Oncotarget. 2017; 8(48)83563-9. 10.18632/oncotarget.18745 PMC5663536 29137364

[35] Ancuta P, Wang J, Gabuzda D (2006). CD16+ monocytes produce IL-6, CCL2, and matrix metalloproteinase-9 upon interaction with CX3CL1-expressing endothelial cells. J Leukoc Biol.

[36] Lee SJ, Namkoong S, Kim YM, Kim CK, Lee H, Ha KS, Chung HT, Kwon YG, Kim YM: Fractalkine stimulates angiogenesis by activating the Raf-1/MEK/ERK- and PI3K/Akt/eNOS-dependent signal pathways. Am J Physiol Heart Circ Physiol 2006, 291(6):H2836-46. 10.1152/ajpheart.00113.2006 16877565

[37] Robinson JD, Pitcher JA (2013). G protein-coupled receptor kinase 2 (GRK2) is a Rho-activated scaffold protein for the ERK MAP kinase cascade. Cell Signal.

[38] Filardo EJ, Quinn JA, Frackelton AR, Bland KI (2002). Estrogen action via the G protein-coupled receptor, GPR30: stimulation of adenylyl cyclase and cAMP-mediated attenuation of the epidermal growth factor receptor-to-MAPK signaling axis. Mol Endocrinol.

[39] Shakibaei M, John T, Schulze-Tanzil G, Lehmann I, Mobasheri A (2007). Suppression of NF-kappaB activation by curcumin leads to inhibition of expression of cyclo-oxygenase-2 and matrix metalloproteinase-9 in human articular chondrocytes: implications for the treatment of osteoarthritis. Biochem Pharmacol.

[40] Tong KM, Chen CP, Huang KC, Shieh DC, Cheng HC, Tzeng CY, Chen KH, Chiu YC, Tang CH (2011). Adiponectin increases MMP-3 expression in human chondrocytes through AdipoR1 signaling pathway. J Cell Biochem.

[41] Liu FL, Chen CH, Chu SJ, Chen JH, Lai JH, Sytwu HK, Chang DM (2007). Interleukin (IL)-23 p19 expression induced by IL-1beta in human fibroblast-like synoviocytes with rheumatoid arthritis via active nuclear factor-kappaB and AP-1 dependent pathway. Rheumatology (Oxford).

[42] Montaseri A, Busch F, Mobasheri A, Buhrmann C, Aldinger C, Rad JS, Shakibaei M (2011). IGF-1 and PDGF-bb suppress IL-1beta-induced cartilage degradation through down-regulation of NF-kappaB signaling: involvement of Src/PI-3 K/AKT pathway. PLoS One.

